# EMA-based fitting of optical transitions in CoPc: insights into carrier dynamics

**DOI:** 10.1039/d5ra06277b

**Published:** 2025-10-10

**Authors:** Nawal K. Almaymoni, Shoug M. Alghamdi, Ahmed S. Abdo, Wafaa B. Elsharkawy, Taghreed Alsulami, Maged S. Al-Fakeh, Ayman M. Mostafa, A. Abouelsayed

**Affiliations:** a Department of Physics, College of Science, Princess Nourah Bint Abdulrahman University P.O. Box 84428 Riyadh 11671 Saudi Arabia; b Department of Physics, College of Science, Taibah University Yanbu Governorate Saudi Arabia; c Department of Physics, Egyptian Customs Authority Cairo Egypt; d Physics Department, College of Science and Humanities, Prince Sattam Bin Abdulaziz University Alkharj 11942 Saudi Arabia; e Department of Physics, Faculty of Science, Umm Al-Qura University Makkah 24382 Saudi Arabia; f Department of Chemistry, College of Science, Qassim University Buraidah 51452 Saudi Arabia; g Department of Physics, College of Science, Qassim University P. O. Box 6644, Almolaydah Buraydah 51452 Saudi Arabia a.mostafa@qu.edu.sa; h Spectroscopy Department, Physics Research Institute, National Research Centre 33 El Bohouth st., P.O. 12622 Dokki Giza Egypt as.abouelsayed@gmail.com

## Abstract

Optical measurements have been performed in a wide frequency range between 400 cm^−1^ and 52 000 cm^−1^ on partially oxidized cobalt phthalocyanine (CoPc). The mid-IR band for the electric field E parallel to the stacking axis, centered at about 4008 for [CoPc]^+^ ions, respectively, has been discussed in terms of resonance absorption based on the effective medium approximation (EMA) model. The band in the reflectivity and conductivity spectra has been fitted using the normal Lorentz function and the general form of the Maxwell–Garnett equation. Various fitting features, which are explored for free electron metals, are obtained by inserting one Drude term in the Maxwell–Garnett equation, such as the plasma frequency and the time scattering rate. Based on the parameters extracted from fitting the mid-IR band according to the EMA model, we were able to obtain the effective conductivity *σ*_eff_ (*ω*) as a function of frequency.

## Abstract 2

Effective medium approximation (EMA) fitting of mid-infrared optical transitions in partially oxidized cobalt phthalocyanine (CoPc) reveals anisotropic carrier dynamics governed by molecular stacking and electronic structure.

## Graphical abstract

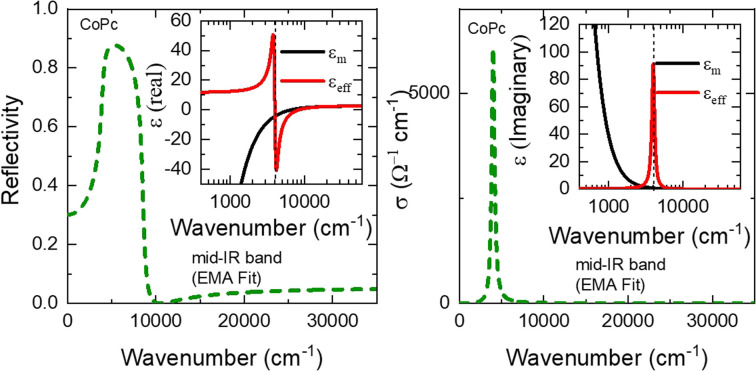

## 1 Introduction

Cobalt phthalocyanines (CoPc) are considered to be one of the important species that have been used in many industrial applications for half a century.^1,2^ What has attracted researchers' interest in MPcs materials is their presence in the form of planar macrocyclic ring compounds, which also give them distinctive physical and chemical properties. Therefore, many different experimental studies have been conducted on them to study their optical, electrical, and magnetic properties.^3–5^ While each MPc molecule is a two-dimensional, nearly planar macrocycle, the crystalline solid often adopts a one-dimensional columnar stacking motif. In these structures, the planar molecules stack along a crystallographic axis with their molecular planes oriented nearly perpendicular to the stacking direction. Such columnar packing imparts anisotropic electronic and transport properties. The crystalline structures of CoPc have been determined experimentally and reported in detail in previous studies.^6–9^ The electrical and optical properties of the crystalline CoPcs are dependent on the degree of oxidation of a macrocyclic ligand (Pc) and the type of the central Co. In principle, MPcs can be reversibly oxidized and reduced both chemically and electrochemically. Removal of one electron from the neutral species generates the radical cation [MPc]^+^. Conversely, stepwise addition of one, two, three, or four electrons leads to the monoanion [MPc]^−^, the dianion [MPc]^2−^, the trianion [MPc]^3−^, and the tetraanion [MPc]^4−^, respectively.^10,11^ One-electron oxidation in MPcs occurs from the Pc ring a_1u_ orbital, not the metal d orbital. In most transition metals as a central ion, the a_1g_ and e_g_ orbitals lie above a_1u_.^12^ Partially oxidized Pc rings in NiPcI, NiPc(AsF_6_)_0.5_, PtPc(ClO_4_)_0.5_, PtPc(AsF_6_)_*x*_, and H_2_PcI behave as one-dimensional metals at high temperatures.^13–16^ All conductive MPc compounds crystallize in a tetragonal system, in which the molecules are arranged in one-dimensional stacks with a metal-over-metal overlap. This packing facilitates efficient orbital overlap along the stacking direction, promoting charge transport and high conductivity within the columnar structure.^13^ This group includes non-magnetic phthalocyanines (NiPc, PtPc, H_2_Pc) that maintain high conductivity at low temperatures without a Peierls transition.^14–16^ However, the H_2_Pc(AsF_6_)_0.67_ and LiPc, which belong to triclinic and tetragonal crystal structures, respectively, show a semiconductive property.^13^ The second group includes magnetic phthalocyanines (CoPc, CuPc) with central-metal magnetic moment.^17–23^ CuPcI has metal-like behavior,^18,22^ while CoPcI and CoPc(AsF_6_)_0.5_ already exhibit semiconducting behavior near room temperature.^24^ This is primarily due to the different conduction pathways of CoPcI salt compared to other MPcI salts. Conductive MPcI salts generally have two conduction pathways, either *via* the M^2+^ ions chain or *via* the oxidized Pc(1^−^) chain, depending primarily on their M^2+^Pc(1^−^)-on-M^2+^Pc(1^−^) stacking structure. Thus, both become conductive pathways. In almost all MPcI metal salts, the Pc molecule is oxidized and acts as a conduction pathway, while in CoPcI salt, the Co^2+^ ions are responsible for conduction.^25^ The strong Coulomb interaction between the electrons of Co^2+^ ions creates the semiconducting property. The interpretation of the optical spectra of the oxidized MPcs has been the subject of experimental investigations.^1,24^

Guruswathi *et al.*^26^ recently conducted optical and photoelectrical studies on evaporated CoPc thin films, highlighting how film morphology and substrate interactions influence absorption features and charge transport. The structural distortions and substrate interactions have been shown to modify the electronic structure of CoPc, with consequences for optical and catalytic properties as stated by Yang *et al.*^27^ For general background on recent developments in phthalocyanine-based hybrids and their optoelectronic characterization, see the recent review by Puttaningaiah.^28^ Recent perspectives summarize advances in the electronic and optical characterization of transition-metal phthalocyanines and provide context for comparisons across different central metals.^29^

There are different, distinct features in the reflectivity and conductivity spectra of MPcs with respect to and when the alternating electric field *E* is parallel to the stacking axis or perpendicular to it. For *E*_⊥_ to the stacking axis (field parallel to the molecular plane), the interband transitions observed in an isolated molecule are largely retained in the solid state because the chemical bonds within the molecule are much stronger than the weak van der Waals forces between molecules. As a result, the electronic structure of each molecule remains essentially intact in the solid, allowing its characteristic optical transitions to persist. The oxidation analysis of a free molecule is thus applicable to the solid for *E*_⊥_ stacking axis. Yakushi *et al.*^13^ discussed the optical spectrum of NiPc(AsF_6_)_0.5_ in compression with the solution spectrum of LuPc_2_, which is regarded as a phthalocyanine neutral-radical dimer composed of oxidized Pc(1^−^) and unoxidized Pc(2^−^) components, Pc(1^−^)Lu(3^+^)Pc(2^−^). For *E*_∥_ to the stacking axis (the field is perpendicular to the plane of the molecules), free electron Drude features appeared in the optical spectra, such as in NiPc(AsF_6_)_0.5_, which is formally viewed as an infinite chain of alternating radical NiPc(1^−^) and neutral NiPc(2^−^).

Furthermore, Yakushi *et al.*^13^ discussed the strong mid-IR band at 3700 cm^−1^ in the *E*_∥_*c* spectrum for H_2_Pc(AsF_6_)_0.67_ as a charge-transfer transition between the trimer structures. However, the mid-IR peak at 4800 cm^−1^ for LiPc and CoPc(AsF_6_)_0.5_ is explained in terms of interband transition across the Mott–Hubbard gap.^13,24,30^ In principle, the optical properties of partially oxidized CoPc are somewhat different from those of MgPc, because the central metal of Co atoms contains 3d^7^ electrons. One of the fundamental differences between the reflectivity spectra of MgPc and CoPc lies in the presence of a partially filled 3d level, which leads to the formation of many covalent bonds using different levels of Pc molecules. In fact, the a1u is purely associated with Pc, and its energy is unaffected by metal substitution, where a_1u_ (π) and e_g_ (π*) are the HOMO and LUMO of the Pc ring, respectively.^12^ The MO ordering in MPc reflects the symmetry-allowed overlap between the central metal 3d orbitals and the π orbitals of the macrocyclic ring: substantial. d_*x*^2^−*y*^2^_–π antibonding interactions typically raise this orbital in energy, while the d_*z*_^2^ remains lower due to weaker overlap. This interaction pattern underlies the characteristic electronic structure of MPcs and varies systematically with the metal ion.

In this paper, we have discussed the mid-IR band using the EMA model of a one transition-metal phthalocyanine, CoPc, with 3d^7^ electrons. The mid-IR band is discussed in terms of resonance absorption based on the EMA model. This band in the reflectivity and conductivity spectra are fitted by using the general form of Maxwell–Garnett equation, which expresses the relationship between the effective dielectric constant *σ*_eff_ (*ω*) of the materials, and the depolarization factor g of the metal particles, considering that the metal particles are dispersed in a polarizable insulator of a relative dielectric constant *ε*_T_ rather than in vacuum. By fitting the mid-IR band with the EMA model, we were able to extract several key parameters typically associated with free-electron metals, such as the plasma frequency and scattering time. The high-conductivity resonance at a few eV is unlikely from standard free-electron intraband transport and is better interpreted as a transverse plasma resonance. In this system, light propagates through stacked molecular layers oriented perpendicular to the stacking axis, with interlayer separation enabling a response to the transverse electric field. The EMA-based fitting of the mid-IR band thus provides insight into the scattering time and the frequency-dependent number of collisions along the stacking axis.

## 2 Experimental

The starting powdered materials were obtained from Fluka Chemica company with a purity of about 97.99%. As previously explained elsewhere, films from powdered samples of CoPc were prepared for optical measurements.^31,32^ An appropriate amount of powdered crystals was mixed with 50 mL of distilled water. The solution was sonicated for 30 min using a VCX 750 tip sonicator (13 mm probe). A vacuum filtration system was employed to prepare thin films from the samples on cellulose nitrate membranes. The films were thoroughly washed with distilled water and then dried at 50 °C for 3 h to obtain suitable samples for XRD and optical measurements. The crystalline structure was analyzed using XRD, with diffraction patterns recorded in the 2*θ* range of 10–80° on a Bruker D8 Advance diffractometer operating in continuous scan mode with a step size of 0.01° and a scan rate of 4 min^−1^. Infrared transmission spectra were collected in the 400–4000 cm^−1^ range using a VERTEX 80v spectrometer (Bruker Co., Germany) equipped with a HYPERION 2000 infrared microscope, a 15× Cassegrain objective, and an MCT detector. The spectrophotometer used is a vacuum-type interferometer. An appropriate amount of powdered crystals was mixed with a KBr powder to form a pellet for the transmission measurements. The electronic valence states and surface elemental composition were analyzed using a SPECS X-ray photoelectron spectrometer (XPS). Reflectivity measurements were performed in the 190–2500 nm range with a JASCO V-770 UV-vis spectrophotometer. Reflectance spectra were calculated using barium sulfate as the reference mirror.

## 3 Results and discussion

**Fig. 1:**
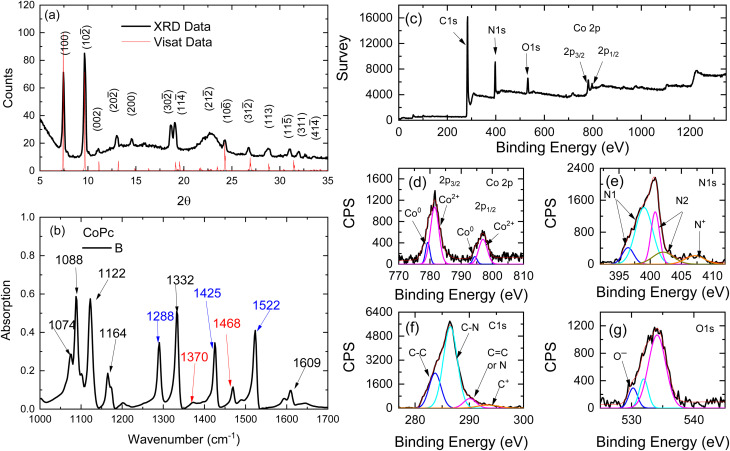
(a) XRD pattern of the monoclinic CoPc film, (b) FTIR absorption spectrum of powdered samples of CoPc, the oxidized and unoxidized characteristic vibrational bands of phthalocyanine rings are denoted by red and blue arrows, respectively, (c) XPS survey spectrum of CoPc sample. High-resolution XPS spectra of (d) Co 2p, (e) N 1s, (f) C 1s and (g) O 1s.

**Fig. 2:**
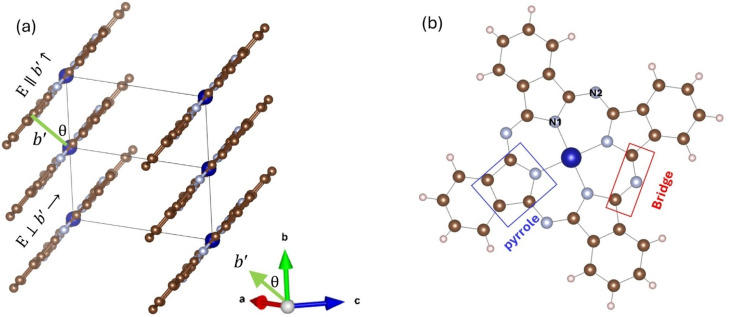
(a) The crystal structure of the monoclinic CoPc, the ranges of fractional coordinates are one unit along the *x*-axis, two units along the *y*-axis, and zero units along the *z*-axis. (b) The molecular scheme of metal phthalocyanines.

The XRD pattern of CoPc film is shown in Fig. 1(a). The XRD patterns of CoPc were indexed to pure monoclinic structure with space group *P*2_1_/*c* and the lattice parameters are *a* = 13.92 Å, *b* = 4.798 Å, *c* = 18.577 Å, and *β* = 121.3°. The Vesta software was used to build up the crystal structure of the CoPc sample according to the reported data in the literature.^9^ The red curve in Fig. 1(a) shows the calculated XRD pattern from Vesta software. The crystal structure is shown in Fig. 2(a). The most intense Bragg peaks are shown in Fig. 1(a).

Fig. 1(b) shows the FTIR spectrum of the CoPc powdered sample. In general, spectroscopic measurements reveal that the symmetry of MPc in solution is *D*_4h_.^3,30^ IR studies have shown that structural distortions and substrate interactions can significantly modify the electronic and vibrational signatures of CoPc.^33,34^ The MPc molecule consists of a macrocyclic ligand (Pc) and central metal (M) atoms. In MPc, the central metal (M) is coordinated in square-planar geometry by four nitrogen donors of the macrocyclic ring. The M–N bond lengths depend strongly on the ionic radius and electronic configuration of M, and this geometric adjustment propagates to the N–C bonds of the phthalocyanine framework, subtly tuning the electronic conjugation of the macrocycle.^12^ The bond lengths between the N atoms and the carbon C atoms within the Pc ring are also dependent on the type of central metal. The molecular structure of MPc is illustrated in Fig. 2(b). The IR spectrum of the CoPc sample agrees very well with the data reported in the literature.^24^

The diagnostic peaks for the CoPc sample are marked with arrows on this spectrum. The intensive peak at 1522 cm^−1^ is attributed to the displacement vibrations of C–N–C bridge bonds of phthalocyanine macrocycle.^35^ The peaks observed at 1288 cm^−1^ and 1425 cm^−1^ correspond to the stretching vibrations of the C–N and C–C bonds, respectively, within the pyrrole rings. These vibrations reflect the characteristic bonding and structural framework of the pyrrole moieties in the molecule.^36^ The two weak absorption peaks at 1609 cm^−1^ and 1468 cm^−1^ are attributed to the stretching vibrations of the C=C bonds in the benzene rings, reflecting the delocalized π-bonding within the aromatic system.

Additionally, the C–N stretching vibration of the pyrrole ring, coordinated to the central Co atom, is observed at 1074 cm^−1^, indicating the interaction between the metal center and the ligand framework. The bending vibrations of the C–H bond in the plane of the molecule appeared at 1088 cm^−1^, 1122 cm^−1^, and 1164 cm^−1^.^36^ For neutral Co(ii)Pc(2^−^), three characteristic FTIR bands are observed at 1288 cm^−1^, 1425 cm^−1^, and 1522 cm^−1^, whereas the partially oxidized Co(ii)Pc(1^−^) exhibits two diagnostic bands at 1370 cm^−1^ and 1468 cm^−1^. As illustrated in Fig. 1(b), the intensity ratio between the oxidized and unoxidized bands can be used to quantify the degree of oxidation within the phthalocyanine chains. The presence of both sets of bands in our FTIR spectra indicates that a certain fraction of the CoPc molecules in the chains has undergone oxidation, confirming partial oxidation of the material.

Fig. 1(c) shows the XPS survey scan spectrum of the CoPc film. Obviously, one can identify the Co, N, C, and O elements from the XPS survey scan spectrum. The O 1s peak might be due to the interaction of H_2_O molecules with the CoPc molecules during the sonication using a tip sonicator for 30 min for film preparation. Fig. 1(d) shows the high-resolution spectrum of Co 2p, which is characterized by two components Co 2p_3/2_ and Co 2p_1/2_, appearing because of spin–orbital splitting. The two peaks at 779.2 eV and 794.3 eV are due to Co^0^ 2p_3/2_ and Co^0^ 2p_1/2_, respectively. The spin–orbital splitting between the Co^0^ 2p_3/2_ and Co^0^ 2p_1/2_ peaks is 15.1 eV. The other satellite peaks at 781.5 eV and 797 eV are due to Co^2+^ 2p_3/2_ and Co^2+^ 2p_1/2_, respectively. The spin–orbital splitting between Co^2+^ 2p_3/2_ and Co^2+^ 2p_1/2_ peaks is 15.9 eV. The high-resolution spectrum of the N 1s is shown in Fig. 1(e). In the spectrum, the two peaks at 397 eV and 398.8 eV are related to N1 atoms, where N1 atoms form the bonds N–C and N–Co in pyrrolic structure in CoPc molecules, while the two peaks at 400.9 eV and 402.1 eV are related to N_2_ atoms for the bridge structures of C–N=C in analogies to NiPc molecules.^37^ The peak that appeared at 407.15 eV can be related to the oxidized N atoms C–N^+^, which is an indication of a certain oxidation of the pc ring. The C 1s spectrum for the CoPc sample is shown in Fig. 1(f). The spectrum was deconvoluted into four peaks at 283.6 eV, 286.3 eV, 290.15 eV, and 293.7 eV. The prominent peaks at 283.6 eV and 286.3 eV can be assigned to the carbon atoms in the phenyl rings and pyrrole units, respectively. These peaks correspond to the C–C bonds in the aromatic phenyl groups and the C–N bonds within the pyrrole rings, reflecting the distinct chemical environments of the carbon atoms in the molecular framework. The peak at 290.15 eV is likely associated with the π–π* satellite.^38^ The peak that appeared at 293.7 eV is related to the oxidized C^+^ atoms, which are also an indication of a certain oxidation of the pc ring. The O 1s binding energies appeared at 531 eV to 534.4 eV, where OH^−^ is at 530.3 eV.

**Fig. 3:**
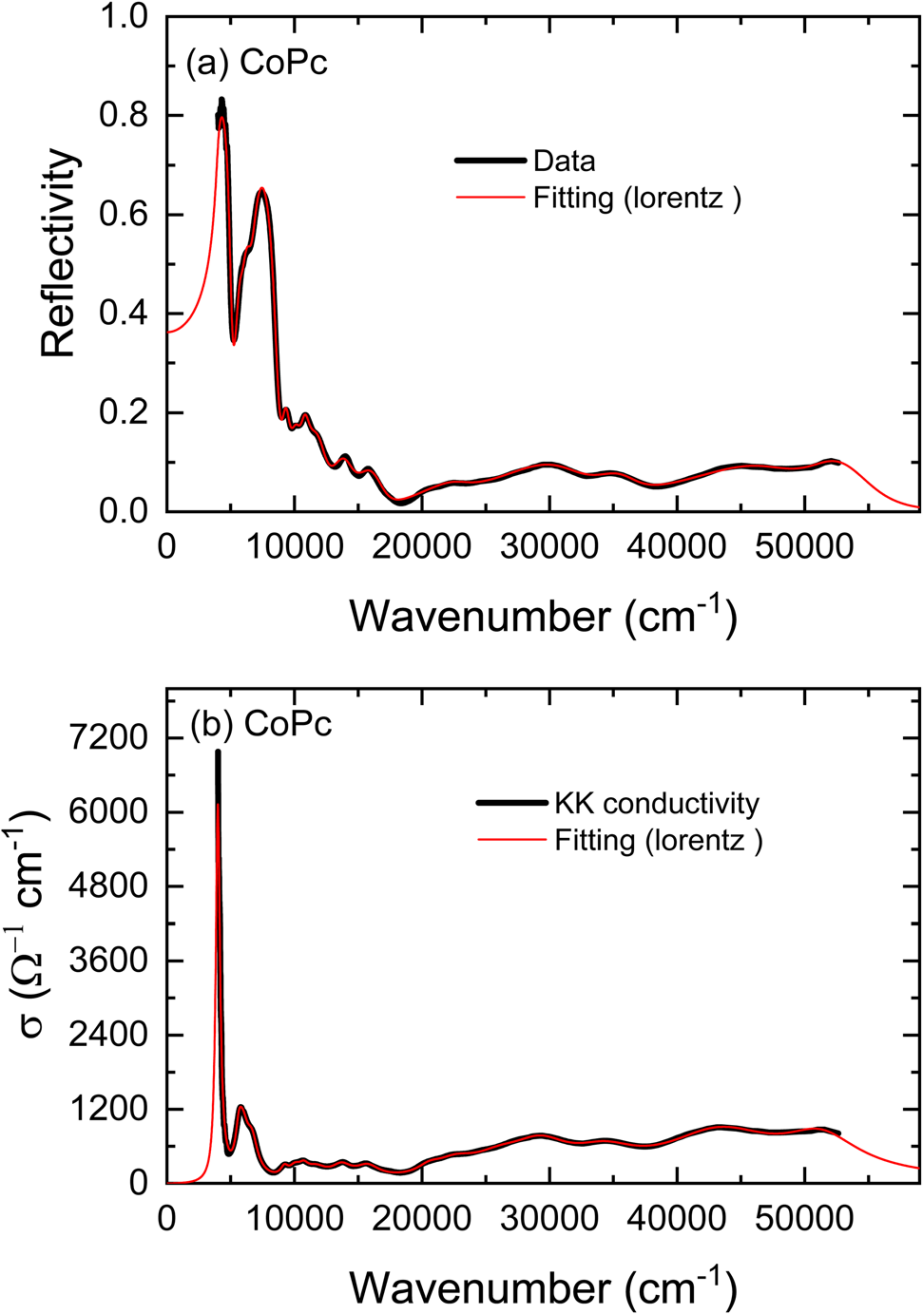
(a) The reflectivity spectrum together with a fitting curve by using a sum of Lorentz oscillators for CoPc. (b) The optical conductivity spectra derived from the KK analysis and those obtained from fitting the reflectivity spectrum.

The unpolarized reflectivity spectrum for CoPc films is presented in Fig. 3(a). The monoclinic crystal structure of CoPc does not show any isotropic properties along any of the principal crystal axes *a*, *b*, or *c*-axis, in contrast to the tetragonal crystal of CoPc, where the conductivity is isotropic along the principal crystal axes.^24^ The conductivity shows a strong mid-IR band along the *c*-axis, and some other interband transitions along the *a*–*b*-axis. However, if we consider the *b*′ axis makes an angle *θ* with the main *b*-axis, where the length of the *b*′ axis is *b* cos *θ*. The *b*′ is the shortest distance between the molecules (see Fig. 2(a)). Yanagisawa *et al.*,^39^ studied the band dispersion of the monoclinic ZnPc along the molecular stacking direction (*b*-axis) as a function of the tilt angle *θ*. They figured out that the distance between the molecules and the molecule tilt angle are important factors that influence the electronic band dispersion. We could analyze the unpolarized reflectivity in terms of *E*_∥_*b*′ axis, and *E*_⊥_*b*′ axis (see Fig. 2(a)).

The reflectivity spectrum includes a strong mid-IR band at around 4008 cm^−1^, which can be considered as a result of the interaction of electromagnetic waves with CoPc molecules, for *E*_∥_*b*′ axis, *i.e.*, the electric field is perpendicular to the plane of the molecules. The reflectivity spectrum also includes various bands at around 10 521 cm^−1^, 13 800 cm^−1^, 20 500 cm^−1^, and 29 100 cm^−1^. Those bands are considered to be a result of the interaction of electromagnetic waves with CoPc molecules, for *E*_⊥_*b*′ axis, *i.e.*, the electric field is parallel to the plane of the molecules. Spectral analysis was performed by estimating the optical conductivity spectrum for CoPc film from the measured reflectivity spectra using Kramers–Kronig (KK) analysis, as follows.

The measured reflectivity spectra span only a limited frequency range; thus, for KK analysis, extrapolation to both lower and higher frequencies is required. To address this, we applied an approximate fitting procedure to the reflectivity data *R*(*ω*). Most of the observed spectral features exhibit symmetric line shapes, which can be effectively modeled using a simple three-parameter Lorentz oscillator. In this framework, the dielectric function is expressed as:^40,41^1
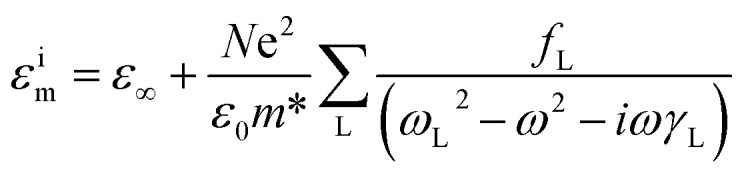
*n* these equations, *ω*_L_, *f*_L_, and *γ*_L_ represent the resonance frequency, oscillator strength, and FWHM of each resonance band, respectively. The parameter *ε*_∞_ denotes the optical dielectric constant at frequencies well above the resonances. The complex reflection *r̃*(*ω*) and the complex refractive index *ñ*(*ω*) are mathematically linked to the experimentally measured reflectivity through the following relation. This connection allows the optical constants of the material to be derived from the reflectivity data, providing insight into its dielectric and dispersive properties.^42^2
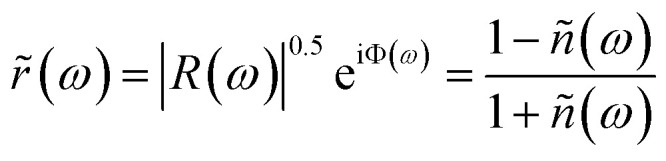


The measured reflectivity spectrum was fitted precisely by using eqn (1) and (2). The result of the fitting curve together with the measured reflectivity spectrum of CoPc is shown in Fig. 3(a). The fitted curve was used to extrapolate the experimental spectrum to lower and higher frequencies for KK analysis. The KK relation for the reflectivity phase *R* is given by:^40^3
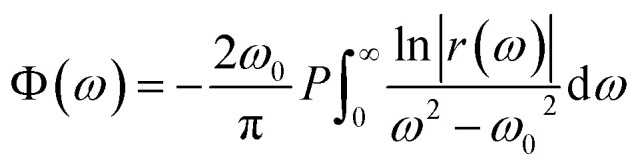


The aim is to obtain the real part of the optical conductivity, which describes the absorption of light in matter, from the fitting parameters of the reflectivity spectrum according to eqn (1) and (2) and then comparing it to that estimated from the KK analysis. The complex optical conductivity *σ̃*(*ω*) is related to the complex dielectric constant *ε̃*(*ω*) according to the following equation:^40,41^4
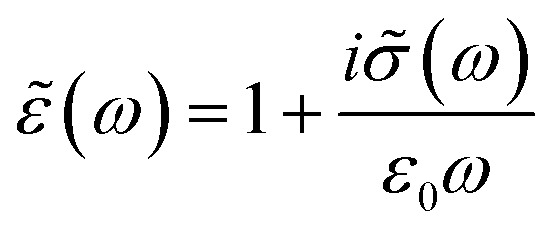


The reliability of the calculation is assessed by comparing the optical conductivity derived from KK analysis with that obtained through fitting of the reflectivity spectrum. As an example, Fig. 3(b) presents this comparison for the CoPc sample, showing an excellent agreement between the two spectra. The fitting parameters used to obtain the conductivity spectrum from the reflectivity data were *ω*_0_ = 4008 cm^−1^, *γ*_L_ = 390 cm^−1^ and *ε*_∞_ = 2.4.

**Fig. 4:**
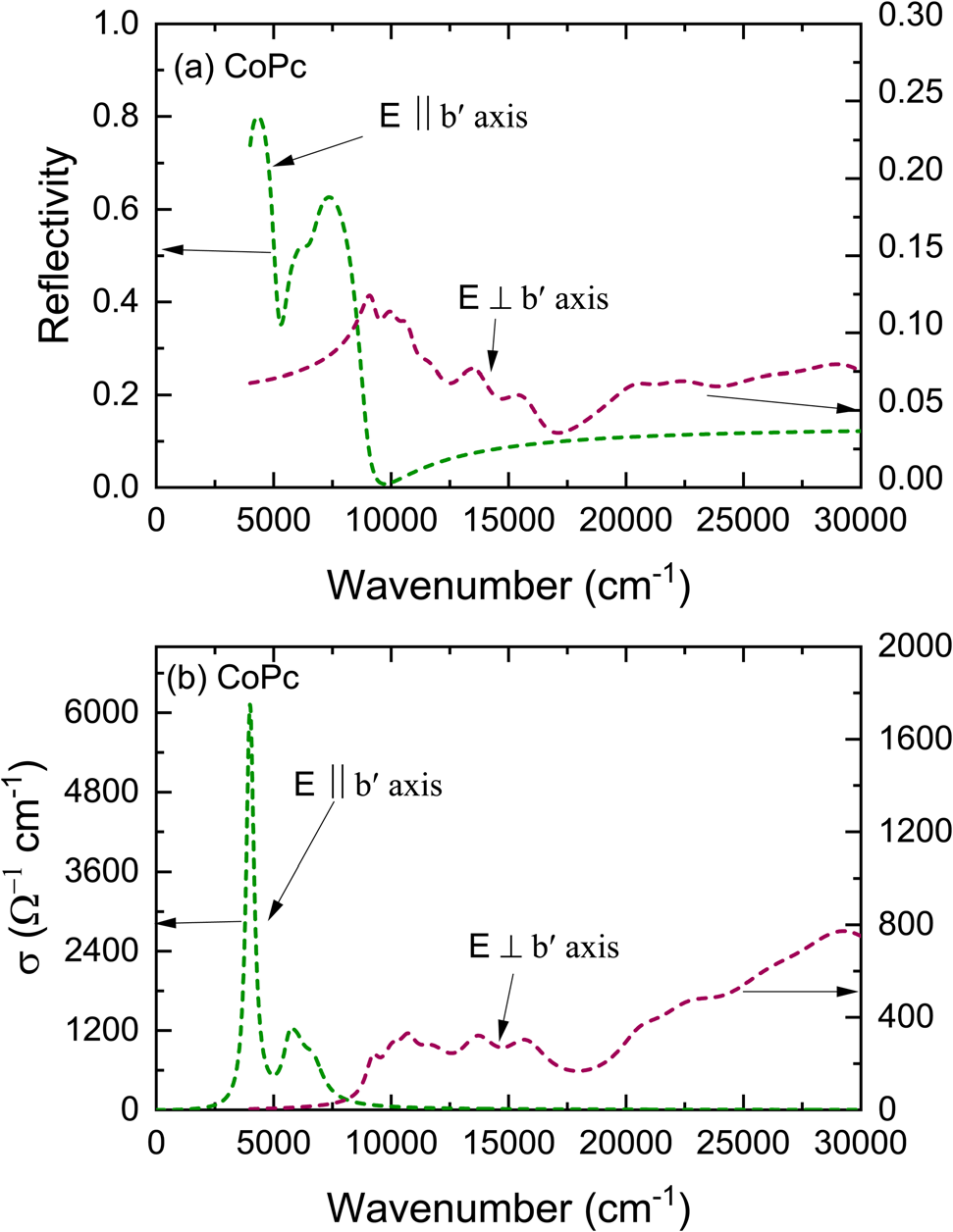
(a) The deconvoluted spectra extracted from fitting the unpolarized reflectivity spectrum by considering that the mid-IR band at 4008 cm^−1^ is related to *E*_∥_*b*′ axis and the higher interband transition above 8500 cm^−1^ is related to *E*_⊥_*b*′ axis. (b) The related deconvoluted spectra of the conductivity spectra for *E*_∥_*b*′ axis and for *E*_⊥_*b*′ axis.

**Fig. 5:**
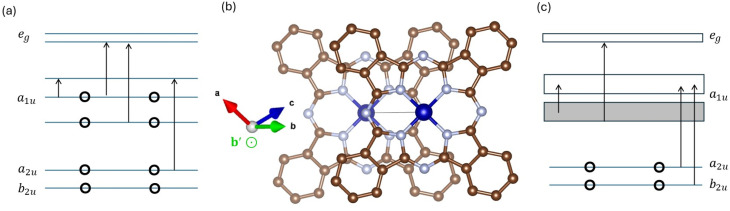
Schematic drawing of the electronic transitions (a) mimic the electronic transition in case of the tetragonal crystal of H_2_Pc(AsF_6_)_0.67_ sample, which considered as a trimer of phthalocyanine (radical-neutral-radical),^13^ (b) The crystal structure of the monoclinic CoPc, the overlay of two formula unit in the *b*′ direction, (c) mimic the electronic transition in case of LiPc sample, which explained in terms of interband transition across the Mott–Hubbard gap.^13^

The deconvoluted spectra extracted from fitting the unpolarized reflectivity spectrum are shown in Fig. 4(a) and (b). For *E*_∥_*b*′ spectrum, the conductivity spectrum includes a strong mid-IR band at around 4008 cm^−1^ and one more band at 5800 cm^−1^. In comparison with the results obtained by Yakushi *et al.*,^13^ the tetragonal crystal of H_2_Pc(AsF_6_)_0.67_ is considered as a trimer of phthalocyanine (radical-neutral-radical) composed of one unoxidized Pc(2^−^) and two oxidized Pc(1^−^) components on the form of Pc(l^−^)Pc(2^−^)Pc(1^−^). The optical conductivity spectrum of isotropic H_2_Pc(AsF_6_)_0.67_ crystals consists of one strong mid-IR band at 3700 cm^−1^ in the *E*_∥_*c* and three bands in the *E*_⊥_*c*. Such transitions are analogous to the electronic transition illustrated in the schematic representation of Fig. 5(a). Thus, the lowest-energy excitation observed at 4008 cm^−1^ in the *E*_∥_*b*′ spectrum can be attributed to a charge-transfer transition involving the phthalocyanine units. Specifically, this transition corresponds to the process Pc(1^−^) + Pc(2^−^) + Pc(1^−^) → Pc(2^−^) + Pc(1^−^) + Pc(1^−^), in which an electron is transferred between adjacent Pc molecules along the stack. This assignment highlights the role of intermolecular charge redistribution in governing the low-energy optical response of the system. It seems that such electronic transitions are not allowed in the monoclinic phase of CoPc with space group *P*2_1_/*c*, because the energetic overlap between the molecular orbital of the Pc ring and the neighbor Pc ring in the *b*′ direction are not allowed, as the Co^2+^ are sit over the N_2_ atom (in the bridge C=N–C) of the neighbor Pc ring along the *b*′ direction (see Fig. 5(b)). However, there might be some other charge transfer that is allowed between the Co^2+^ d_*z*_^2^ orbital and the neighbor Pc orbitals. The non-bonding d_*z*_^2^ orbital has an unpaired electron in Co^2+^. Since the 3d_*z*_^2^ orbital is elongated to the neighboring Pc orbitals, charge-transfer can occur between adjacent Co^2+^ and Pc(1^−^) through the overlap of 3d_*z*_^2^ and Pc orbitals of the type Co^2+^ + Pc(1) → Co^+^ + Pc(0). To transfer an electron from the 3d_*z*_^2^ orbital of one Co^2+^ ion to Pc(1^−^) radical, we must provide an ionization energy, *I*, to remove an electron, and subsequently, the electron affinity, *A*, is recovered when the electron is added to a neighboring Pc(1^−^) ring. The energy cost in this process is: *U*_eff_ = *I* − *A*. If the two Co^2+^ ions and Pc(1^−^) radical are separated ions, the Hubbard *U*_eff_ is the energy required to remove an electron from a Co^2+^ ion (making it Co^3+^) and transferring it to Pc(^−^1) radical (making it Pc(2^−^)). This type of charge transfer can be described by the one-dimensional Hubbard model, where strong Coulomb repulsion splits the system into two bands, resulting in a Hubbard gap. The magnitude of this gap, however, depends on the interplanar distance between CoPc rings. Therefore, the mid-IR peak observed at 4008 cm^−1^ can be attributed to an interband transition across the Mott–Hubbard gap, which arises from the splitting of electronic states due to the effective on-site Coulomb interaction (*U*_eff_). This behavior is similar to that reported for LiPc, indicating that the strong electron–electron repulsion within the molecular stacks governs the low-energy electronic excitations and the optical response of the system.^13,30^ Such transitions are analogous to the electronic transition illustrated in the schematic representation of Fig. 5(c). In general, for typical π-conjugated organic molecules with intermolecular distances of a few angstroms, a typically ranges from 2 to 3 eV.^13^ The effective on-site Coulomb energy (*U*_eff_) and the transfer integral *t*, which are related to the oscillator strength *f*, can be estimated in the framework of the Hubbard model.^43–46^ The oscillator strength of the mid-IR band is obtained from the equation:5
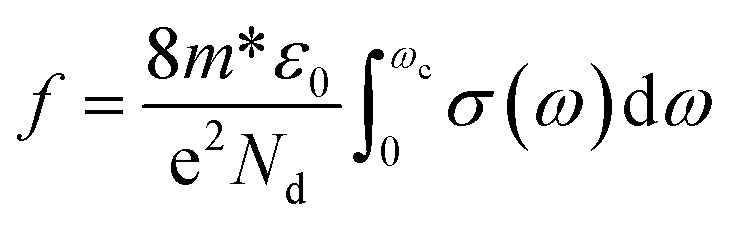
where 
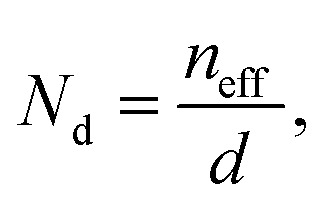
*d* = 4.798 Å is the intermolecular distance in the *b* direction, and *ω*_c_ ≈ 9200 cm^−1^ is the upper limit of the integral. The oscillator strength is found to be *f* = 0.39. By using the experimental values of ECT = 4008 cm^−1^ (0.49 eV), The excitation energy of the mid-IR band, and *f* = 0.39, *d* = 4.4798 Å, the distance between the centers of the molecules, we can estimate *U*_eff_ and *t* to be 0.48 eV, and 0.03 eV, respectively.^43^ The Hubbard bands have a width *W*, and the energy gap *E*_g_ between them is *U*_eff_ − *W*. When this gap is zero, the material becomes metal. Therefore, metallic behavior sets in when *W* − *U*_eff_. The spectral width of the mid-IR band is 4*t* = 0.12 eV (965.3 cm^−1^), which is close to *γ*_L_ The FWHM for the mid-IR band.

Assuming that the upper and lower Hubbard bands have identical bandwidths, each band has a width of 2*t* = 0.06 eV, which is comparable to the bandwidth estimated for monoclinic β-ZnPc.^39^ The value of the bandwidth is substantially lower than the *U*_eff_, and the material is classified as a semiconducting material. The absorption edge of the mid-IR band can be represented by *E*^L^_g_ = *U*_eff_ − *W* = 0.42 eV (3378.6 cm^−1^), which agrees with the observed conductivity spectrum shown in Fig. 4(b). Notice that the hopping integral *t* is inversely proportional to the effective mass. The effective mass estimated for CoPc is about 7.48 me, according to the analysis of the mid-IR band using the EMA model. The smaller the absolute value of the hopping integral, the more difficult it is for the electron to hop from atom to atom, and hence, the larger its effective mass.

Whether we consider that the mid-IR band occurred as a result of the transfer of charges between a trimer of phthalocyanine or as a result of interband transition across the Mott–Hubbard gap, the decay lifetime of electrons can be estimated as follows: The electric dipole matrix element *M*_*ij*_ = 〈*j*|*H*|*i*〉 for a transition from state *j* to *i* is directly proportional to the oscillator strength *f*_*ij*_. The interaction Hamiltonian *H* = −*pE*_0_, where *p* = −*er* is the electric dipole moment of the electron. This matches the expected interaction energy of a dipole in an electric field. *E*_0_ is the electric field strength. For transitions between two bands, the relationship between the matrix element *M*_*ij*_ and the oscillator strength *f*_*ij*_ is given by:^40^6
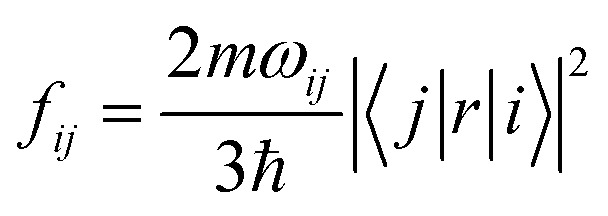
By using *f*_*ij*_ = 0.39 and *ω*_*ij*_ = 4008 cm^−1^ (7.55 × 10^14^ Hz) for the mid-IR band of [CoPc]^+^ ion in eqn (6), the 〈|*j*|*H*|*i*|〉 are found to be 1.1 × 10^−10^. We can use this value to estimate the decaying lifetime. *τ*_*ji*_ of electrons from state *j* to state *i* through the following equation.^41^7
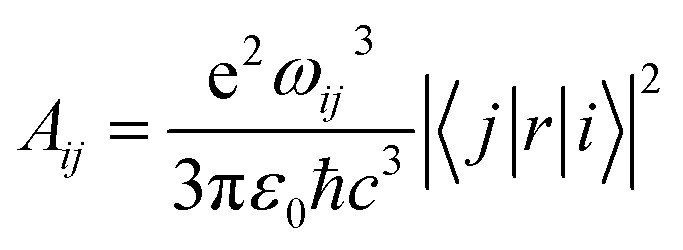
is called the Einstein coefficients for the transition between the conduction and the valence bands. The *τ*_*ji*_ value according to eqn (7) is found to be 1.8 × 10^−6^ s. This decay lifetime is substantially extended, which is related to nonradiative (phonon) recombination. In band-to-band recombination, an electron transitions directly from the conduction band to an unoccupied state (hole) in the valence band. The released energy is either emitted as a photon or transferred to the lattice in the form of thermal vibrations (phonons). Accordingly, band-to-band recombination may be radiative, generally with short radiative lifetimes, typically in the range 10^−9^ – 10^−8^ s and nonradiative or phonon recombination with long radiative lifetimes, typically in the range 10^−6^ s and upwards. With phonon recombination, the excess energy and momentum of an electron are imparted to phonons (lattice vibrations). The maximum energy of a phonon does not exceed 0.1 eV. Therefore, for recombination to take place through a band about 0.49 eV wide, at least 4.9 phonons must be emitted at a time. Indeed, the phonon features appear throughout the mid-IR band in the unpolarized reflectivity and conductivity spectra (this kind of transition might be recalled as a vibronic transition) (see Fig. 3(a) and (b)). We will discuss the average number of collisions of conduction electrons with phonons when we analyze the mid-IR band with the EMA model.

The other bands in the *E*_⊥_*b*′ spectrum are assigned according to the schematic representation shown in Fig. 5(a), in which the electric fields are parallel to the plane of CoPc molecules. The weak absorption band at 10 521 cm^−1^ is ascribed to the transition from the middle a_1u_ (π) to e_g_ (π*). The next band at 13 800 cm^−1^ corresponds to the transition from the lowest a_1u_(π) to the vacant e_g_ (π*). According to Homborg *et al.*,^33^ this transition is referred to as the Q band at about 15 000 cm^−1^ (667 nm). In addition to these central maxima, further weak bands occur in the Q band region, which have been interpreted as vibrational fine structure. The absorption band around 20 500 cm^−1^ is assigned to the transition from the lower filled e_g_ (π) to the top singly occupied a_1u_ (π*). The 20 500 cm^−1^ band cannot be attributed to a vibrational sideband based on its energy and intensity, indicating it is a new electronic transition that vanishes in the neutral molecule. According to Homborg,^33^ the intensity of the Q-band is significantly decreased for the oxidized radical Pc(1^−^) in Mg^2+^Cl(1^−^)Pc(1^−^), and a new band appeared at around 21 000 cm^−1^. Therefore, the band at 20 500 cm^−1^ is interpreted as the electronic transition from the fully occupied e_g_ (π) band to the top empty a_1u_ (π*) band. This interpretation accounts for the disappearance of the 20 500 cm^−1^ band in neutral CoPc and suggests that its presence serves as an indicator of ring oxidation. The intensive band at 29 081 cm^−1^ is called the B band and is assigned to the electron transition a_2u_ (π) to e_g_ (π*) (see Fig. 4(b)).

**Fig. 6:**
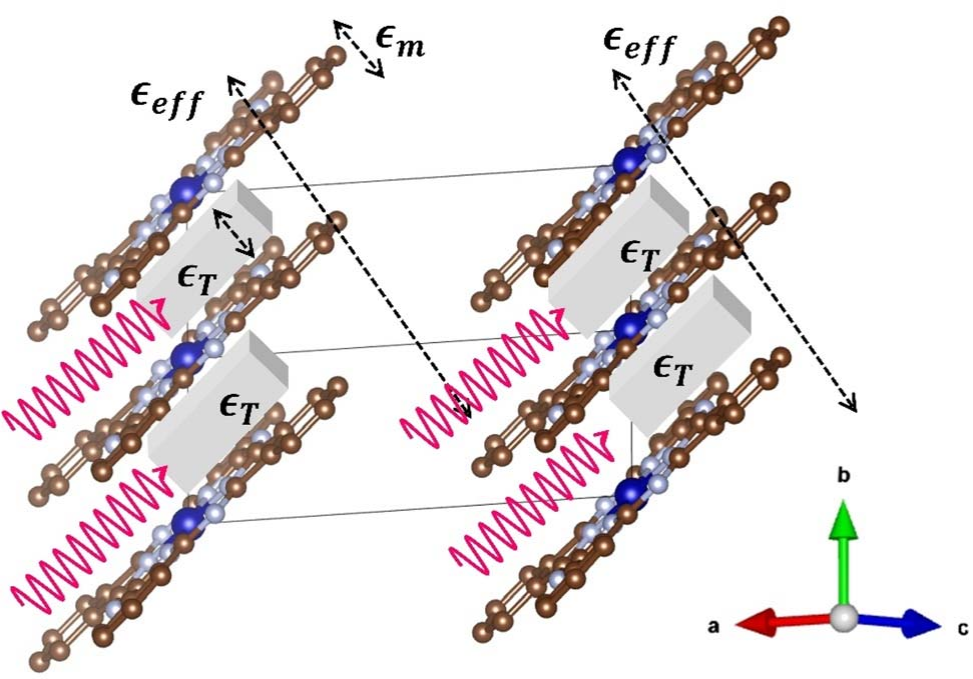
Schematic representation of the unit cell of the CoPc monoclinic crystal structure, representing the way of interacting the electromagnetic waves with CoPc molecules for *E*_∥_*b*′ axis in terms of the EMA model.

The mid-IR band can be further explained in terms of resonance absorption based on the effective medium approximation model. This band in the reflectivity and conductivity spectra can be fitted by using the general form of the Maxwell–Garnett equation, which expresses the relationship between the effective dielectric constant. *ε*_eff_ (*ω*) the materials, the volume fraction *v* of the metal particles and the depolarization factor *g* of the metal particles, taking into account that the metal particles are dispersed in a polarizable insulator of a relative dielectric constant *ε*_T_ Rather than in a vacuum. The most general form of the Maxwell–Garnett equation is as follows:^47^8
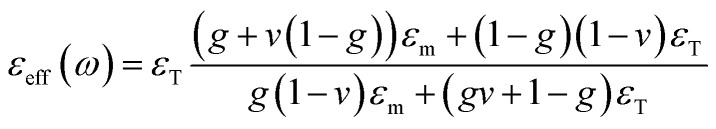
In this equation, we will assume that *ε*_m_ (*ω*) is the dielectric constant of the CoPc molecules, *v* is the volume fraction of the molecules in the dielectric medium (*v* = 1 − *x*), where *x* is the volume fraction of the dielectric medium, and *g* is the depolarization factor. Fig. 6 illustrates the schematic representation of the CoPc monoclinic crystal structure, which represents the way of interacting the electromagnetic waves with CoPc molecules for *E*_∥_*b*′ axis in the framework of the EMA model. To a reasonable approximation, the value of the depolarization factor *g* depends on particle morphology in the dielectric.^47^

**Fig. 7:**
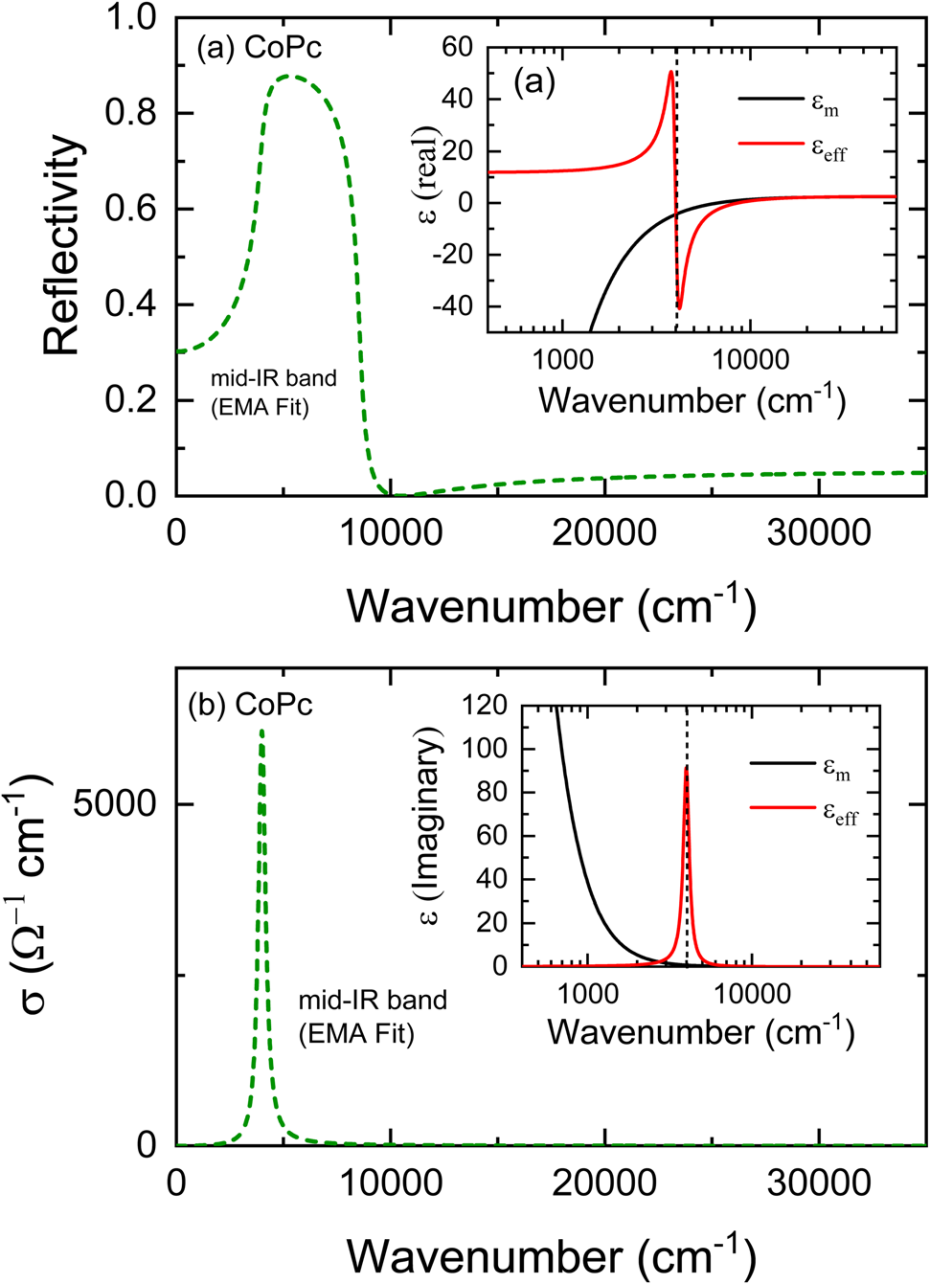
(a) The mid-IR band extracted from fitting the unpolarized reflectivity spectrum by the EMA model (for *E*_∥_*b*′ axis), the inset illustrates the real parts of dielectric constants for free electrons *ε*_m_ and *ε*_eff_ for the collective CoPc molecules. (b) The deconvoluted mid-IR band extracted from the conductivity spectrum for *E*_∥_*b*′ axis, the inset illustrates the imaginary parts of the dielectric constants for free electrons *ε*_m_ and *ε*_eff_ for the collective CoPc molecules.

The *g* values approach zero when the embedded particles are in the form of metal plates with the electric field perpendicular to their vertical axes or when the embedded materials are in the form of long cylinders with the electric field parallel to their axes. In this case, the applied electric field remains unaltered, as no polarization fields are induced outside particles with fields parallel to their surfaces. However, *g* can reach unity when the perpendicular axes of the metal plates align with the field, analogous to a series array of capacitors. In principle, by using the EMA model, one can fit the lower band centered at 4008 cm^−1^ in the reflectivity and conductivity curves by using one Drude term. In the EMA model, the Drude peak of free electrons occurs at a finite frequency, primarily determined by the geometry of molecules in the dielectric, not the charge density.^47,48^ The Drude model is used to describe intraband excitations as a Drude contribution to *R*(*ω*) and *σ*(*ω*) spectra. Specifically, *ε*_m_(*ω*) is decomposed into an interband part *ε*^i^_m_(*ω*) given by eqn (1) and a free-electron part *ε*^f^_m_(*ω*) given through the following equation:^40,41^9
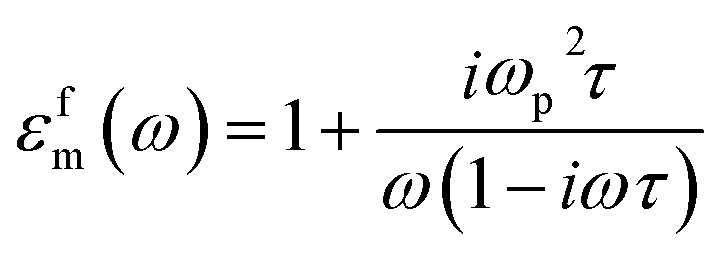
where 
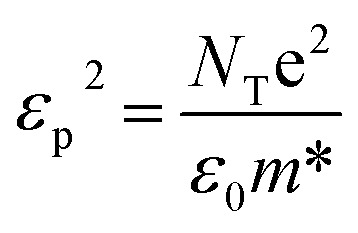
. Here *ω*_p_ Is the free-electron plasma frequency. We used eqn (8)*ε*_m_(*ω*) = *ε*^f^_m_(*ω*) and then fitting the lower band centered at 4008 cm^−1^ in *R*(*ω*) and *σ*(*ω*) spectra based on eqn (2) and (3), and in this case *ε̃*(*ω*) = *ε̃*_eff_ (*ω*), *ñ*(*ω*) = *ñ*_eff_ (*ω*) and *σ̃*(*ω*) = *σ̃*_eff_ (*ω*). Fig. 7(a) and (b) shows the mid-IR band extracted from fitting this band in the reflectivity and conductivity spectra with the EMA model. The values obtained through fitting the lower mid-IR band centered at 4008 cm^−1^ according to the EMA model are *ω*_p_ = 8227.88 cm^−1^, *γ*_D_ = 404 cm^−1^, 
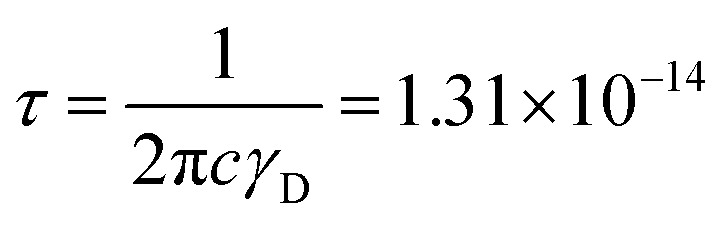
. The values of *ε*_T_ = 1.64, the depolarization factor *g* = 0.88, and the molecules volume fraction *v* = 0.68. For *g* approaches to unity 0.88, it is expected that the at molecules with their normal parallel to the electric field. In this case, the electric fields are parallel to the conducting axis *E*_∥_*b*′. In the provisos study performed with Yakushi *et al.*^48^ in the case of CoPc(AsF_6_)_0.5_, the plasma edge is observed at a significantly higher energy compared to NiPc(AsF_6_)_0.5_. For NiPc(AsF_6_)_0.5_, most of the spectral weight lies below 1500 cm^−1^, whereas the optical conductivity of CoPc(AsF_6_)_0.5_ extends broadly, with a tail reaching up to about 6000 cm^−1^. This behavior suggests that an additional optical transition contributes to the mid-infrared response of CoPc(AsF_6_)_0.5_. The extracted plasma frequencies are 7610 cm^−1^ for NiPc(AsF_6_)_0.5_ and 9590 cm^−1^ for CoPc(AsF_6_)_0.5_. Because both compounds possess the same carrier density (*N*), this difference can be attributed to a variation in the effective mass (*m*). A likely explanation is that the bandwidth is increased in CoPc(AsF_6_)_0.5_ owing to a shorter *c*-axis; the *c*-axis in this compound is approximately 3% smaller than in NiPc(AsF_6_)_0.5_. To explore this effect further, we examined the relationship between plasma frequency and the lattice constant in NiPc(AsF_6_)_0.5_. Recent studies such as Mostafa *et al.*^49^ have characterized the dielectric relaxation and AC conductivity in p-Si/CoPc hybrids, where they examine CoPc molecules under strong coupling to polaritonic modes, shedding light on its vibrational and electronic transitions. These works complement our reflectivity/conductivity spectral analysis by providing insight into the molecular structure and low-frequency electrical behavior. Frequency- and temperature-dependent dielectric and AC conductivity behavior of CoPc has been reported for p-Si/CoPc hybrids, providing experimental benchmarks for low-frequency dielectric relaxation and transport that complement our mid-IR optical results.

Indeed, the phenomenological EMA theory cannot readily explain the conduction resonance. Marton *et al.*^50^ discussed the nature of the conduction absorption band (the mid-IR band in our case) as resonance absorption due to a couple between transverse electromagnetic waves with a collective oscillation of free electrons in all aggregates of metal particles at a particular frequency. Conduction resonance occurs when the real part of the dielectric constant is zero, meaning the local conduction electrons oscillate in phase with the driving electric field (see inset of Fig. 7(a and b)). Therefore, the observed high conductivity at around 0.49 eV is unlikely to originate from a standard intraband transport of free electrons at the Fermi level. Instead, these collective oscillations at this energy can be interpreted as a transverse absorption resonance.^51^

## 4 Conclusion

We studied the wide frequency range unpolarized reflectivity of the partially oxidized monoclinic structure of CoPc with space group *P*2_1_/*c*. FTIR measurements confirm that CoPc samples have a certain amount of oxidation of the Pc ring. The unpolarized reflectivity shows a mid-IR band centered at about 4008 cm^−1^ for [CoPc]^+^ ions, respectively. The band has been considered as a result of the interaction of the electric field with MPcs molecules, where the electric field is incident parallel to the stacking axis of the MPcs molecules. The mid-IR bands have been fitted with a Lorentz function using the EMA model. Through fitting the mid-IR bands with the Lorentz function, we estimated the oscillator strength *f*, the transfer integral *t*, and the effective on-site Coulomb energy *U*_eff_, and the decaying lifetime of electrons from the excited state to the ground state. The decay lifetime estimated for all studied samples for the mid-IR band is found to be long and is related to nonradiative (phonon) recombination. Through fitting the mid-IR bands by using the general form of the Maxwell–Garnett equation, various fitting features are obtained, such as the plasma frequency and the time scattering rate.

## Conflicts of interest

The authors declare no conflict of interest.

## Data Availability

The data supporting the findings of this study are available from the corresponding author upon reasonable request. All relevant experimental data, analysis methods, and modeling results used in this work can be provided to qualified researchers for the purpose of academic and non-commercial investigation.
